# Sensitive and robust gene expression changes in fish exposed to estrogen – a microarray approach

**DOI:** 10.1186/1471-2164-8-149

**Published:** 2007-06-07

**Authors:** Lina Gunnarsson, Erik Kristiansson, Lars Förlin, Olle Nerman, D G Joakim Larsson

**Affiliations:** 1 Department of Neuroscience and Physiology, the Sahlgrenska Academy at Göteborg University, SE-405 30 Göteborg, Sweden; 2 Department of Mathematical Statistics, Chalmers University of Technology, SE-412 96 Göteborg, Sweden; 3 Department of Zoology/Zoophysiology, Göteborg University, SE-405 30 Göteborg, Sweden

## Abstract

**Background:**

Vitellogenin is a well established biomarker for estrogenic exposure in fish. However, effects on gonadal differentiation at concentrations of estrogen not sufficient to give rise to a measurable vitellogenin response suggest that more sensitive biomarkers would be useful. Induction of zona pellucida genes may be more sensitive but their specificities are not as clear. The objective of this study was to find additional sensitive and robust candidate biomarkers of estrogenic exposure.

**Results:**

Hepatic mRNA expression profiles were characterized in juvenile rainbow trout exposed to a measured concentration of 0.87 and 10 ng ethinylestradiol/L using a salmonid cDNA microarray. The higher concentration was used to guide the subsequent identification of generally more subtle responses at the low concentration not sufficient to induce vitellogenin. A meta-analysis was performed with data from the present study and three similar microarray studies using different fish species and platforms. Within the generated list of presumably robust responses, several well-known estrogen-regulated genes were identified. Two genes, confirmed by quantitative RT-PCR (qPCR), fulfilled both the criteria of high sensitivity and robustness; the induction of the genes encoding zona pellucida protein 3 and a nucleoside diphosphate kinase (nm23).

**Conclusion:**

The cross-species, cross-platform meta-analysis correctly identified several robust responses. This adds confidence to our approach used for identifying candidate biomarkers. Specifically, we propose that analyses of an nm23 gene together with zona pellucida genes may increase the possibilities to detect an exposure to low levels of estrogenic compounds in fish.

## Background

The contraceptive estrogen, ethinylestradiol (EE_2_) is an important contributor to the feminization of fish downstream from sewage treatment works [1-5]. This discovery was greatly facilitated by the use of vitellogenin (VTG) as a biomarker. VTG is produced in the liver of sexually maturing female fish under the influence of endogenous estrogen. Normally, VTG is not expressed in males or juveniles, unless they are exposed to estrogens via water or food. Both VTG mRNA and protein in male and juvenile fish have thus become established biomarkers for exposure to environmental estrogens [6]. However, estrogens can effect gonadal sex differentiation of fish at concentration not sufficient to give rise to a measurable VTG response [7]. It has also been shown that life cycle exposure of fathead minnow to an inordinately low concentration of EE_2 _(0.32 ng/L) was sufficient to decrease the egg fertilisation and to skew the sex ratios towards female[8]. This suggests that more sensitive biomarkers would be useful. Zona pellucida (ZP) genes may be more sensitive than VTG [9] but their specificity for estrogens is not as clear [10-12]. Additional, sensitive biomarkers would thus increase our possibilities to identify exposure to low, but biologically important concentrations of estrogens.

Rapidly accumulating data on genomes and proteomes have increased the possibilities to use different types of discovery-driven methods in ecotoxicology [13,14]. The large number of potential responses that can be studied with microarrays renders the method suitable for identifying candidate biomarkers of exposure [15-20]. Such candidates may then be further evaluated to find if they are useful as biomarkers. In general, a good biomarker should be sensitive, specific and robust. A robust response implies for example that it should be measurable at complex exposure situations, at different exposure concentrations, at different temperatures, after different exposure times, by different analytical approaches, in different labs and preferably also in different species.

The main objective of the present study was to use microarrays to find novel, sensitive and robust biomarkers of estrogenic exposure in fish. We have used a salmonid cDNA microarray from cGRASP [21] to analyze hepatic expression profiles in juvenile rainbow trout (*Oncorhynchus mykiss) *exposed to EE_2 _*in vivo*. The responses identified at a high concentration of EE_2 _were used to guide the subsequent identification of generally more subtle responses at a low concentration of estrogen. We also identified estrogen-responses shared between fish species, experimental conditions and analytical platforms. This was achieved by a meta-analysis using our dataset together with results from three recently published articles describing hepatic gene expression profiles in fish exposed to estrogens [16,20,22].

## Results

### Sensitive gene-expression changes

Both male and female juvenile fish exposed to 0.87 ng EE_2_/L were analyzed with microarray. The microarray analysis of female fish suggested that only three out of four females had an induced expression of the known estrogen-responsive gene ZP3. In contrast, an induction was present in all eight males. This observation suggested that some juvenile females may have sufficient endogenous estrogen to induce sensitive estrogen-responsive genes. Thus, in our search for genes responding to low concentrations of estrogens only the microarray results from male fish were used.

**Figure 1:**
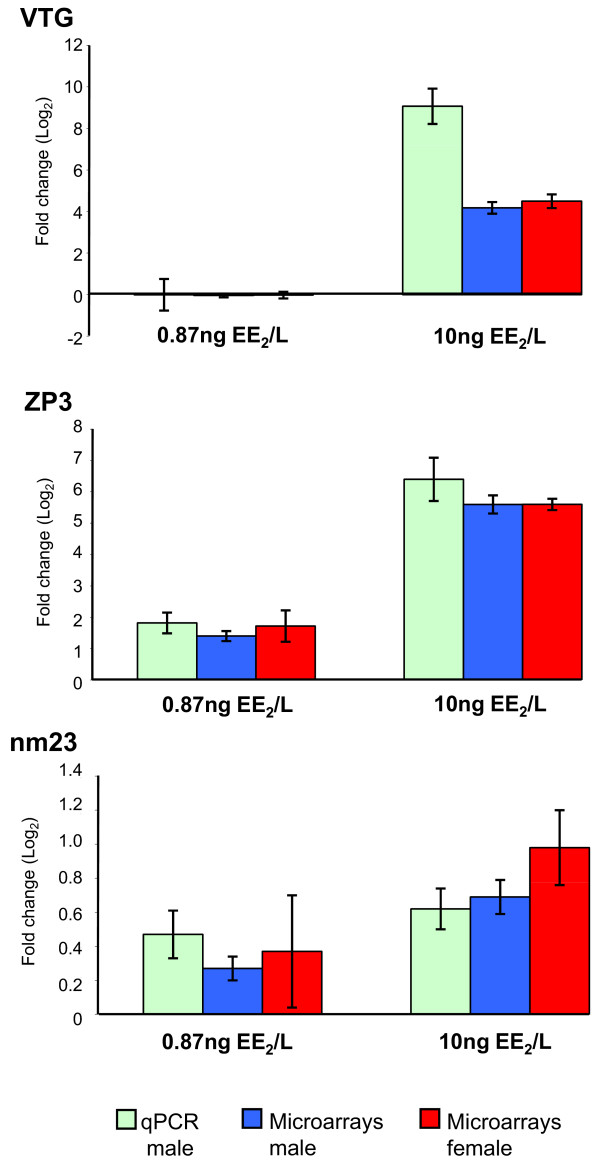
**Gene expression changes of VTG, ZP3 and nm23 measured by qPCR and microarray**. Hepatic gene expression in rainbow trout of vitellogenin (VTG), zona pellucida protein 3 (ZP3) and a nucleoside diphosphate kinase (nm23) after EE_2 _exposure measured with qPCR (green bars, male fish) or microarray (blue bars, male fish: red bars, female fish). Values are expressed as fold change (log_2_) compared to control fish. Paired student's t-tests (single sided) were performed on the qPCR data to confirm/test the putative regulation suggested from microarray data. VTG, ZP3 and nm23 were confirmed to be significantly up-regulated in fish exposed to 10 ng/L (p = 0.001, 0.001 and 0.007 respectively, four biological replicates in each group). ZP3 and nm23, but not VTG were up-regulated in fish exposed to 0.87 ng/L (p = 0.0004, 0.006 and 0.5 respectively, eight biological replicates in each group) in accordance with the microarray data.

Thirty-six sets of cDNAs (presumably corresponding to 29 genes) were regulated in male fish both by the low and the high concentration of EE_2 _(Table 1). All of the cDNAs responded in a dose-dependent manner. ZP3 was the most differentially expressed gene in fish exposed to both high and low concentrations with a fold change of 84 and 3.5 respectively. VTG was not affected by the low concentration while it was up-regulated 537 times by 10 ng EE_2_/L as measured by quantitative RT-PCR (qPCR) (Figure 1).

**Table 1:** Estrogen-sensitive genes.

**cGRASP ID**	**M-value 0.87 ng/L**	**M-value 10 ng/L**	**Annotation**
**CK991165**	**1.40**	**5.60**	**[GO] [P10761] Zona pellucida sperm-binding protein 3 precursor (Zona pellucida glycoprotein ZP3) (Sperm receptor) (Zona pellucida protein C)**,
CB492227	-0.23	-0.34	[GO] [P23506] Protein-L-isoaspartate(D-aspartate) O-methyltransferase (EC 2,1,1,77) (Protein-beta-aspartate methyltransferase) (PIMT) (Protein L- isoaspartyl/D-aspartyl methyltransferase)
CA054422	0.22	0.43	UNKNOWN
CB496664	0.30	0.50	[GO] [Q9D0E1] Heterogeneous nuclear ribonucleoprotein M (hnRNP M),
**CB497378**	**0.27**	**0.69**	**[GO] [P15532] Nucleoside diphosphate kinase A (EC 2,7,4,6) (NDK A) (NDP kinase A) (Tumor metastatic process-associated protein) (Metastasis inhibition factor NM23) (NDPK-A) (nm23-M1)**,
**CB511030**	**0.27**	**0.47**	**[GO] [P15532] Nucleoside diphosphate kinase A (EC 2,7,4,6) (NDK A) (NDP kinase A) (Tumor metastatic process-associated protein) (Metastasis inhibition factor NM23) (NDPK-A) (nm23-M1)**
**CK991305**	**0.27**	**0.59**	**[GO] [Q01768] Nucleoside diphosphate kinase B (EC 2,7,4,6) (NDK B) (NDP kinase B) (nm23-M2) (P18)**,
CA037915	0.30	0.38	[GO] [P35505] Fumarylacetoacetase (EC 3,7,1,2) (Fumarylacetoacetate hydrolase) (Beta-diketonase) (FAA),
CA060608	0.21	0.46	[GO] [P56384] ATP synthase lipid-binding protein, mitochondrial precursor (EC 3,6,3,14) (ATP synthase proteolipid P3) (ATPase protein 9) (ATPase subunit C),
CB496562	0.16	0.32	[GO] [Q9CY58] Plasminogen activator inhibitor 1 RNA-binding protein (PAI1 RNA- binding protein 1) (PAI-RBP1),
CB516182	0.46	0.56	[GO] [O08709] Peroxiredoxin 6 (EC 1,11,1,15) (Antioxidant protein 2) (1-Cys peroxiredoxin) (1-Cys PRX) (Acidic calcium-independent phospholipase A2) (EC 3,1,1,-) (aiPLA2) (Non-selenium glutathione peroxidase) (EC 1,11,1,7) (NSGPx),
**CB511422**	**0.25**	**0.50**	**[GO] [P15532] Nucleoside diphosphate kinase A (EC 2,7,4,6) (NDK A) (NDP kinase A) (Tumor metastatic process-associated protein) (Metastasis inhibition factor NM23) (NDPK-A) (nm23-M1)**,
**CB496931**	**0.80**	**2.58**	**[GO] [P11404] Fatty acid-binding protein, heart (H-FABP) (Heart-type fatty acid- binding protein) (Mammary-derived growth inhibitor) (MDGI)**,
**CB497374**	**0.60**	**2.08**	**[GO] [P11404] Fatty acid-binding protein, heart (H-FABP) (Heart-type fatty acid- binding protein) (Mammary-derived growth inhibitor) (MDGI)**,
CB505692	-0.33	-0.42	UNKNOWN
CB497174	0.53	1.71	[NR] [XP_423045] PREDICTED: similar to nudix (nucleoside diphosphate linked moiety X)-type motif 7; coenzyme A diphosphatase [Gallus gallus]
**CB497649**	**0.21**	**0.52**	**[GO] [Q01768] Nucleoside diphosphate kinase B (EC 2,7,4,6) (NDK B) (NDP kinase B) (nm23-M2) (P18)**,
CA037988	0.23	0.41	[NT] [AJ488155] Pachymedusa dacnicolor partial mRNA for ribosomal protein S16 (rps16 gene)
CB499596	0.14	0.49	[NR] [NP_077217] hydroxysteroid dehydrogenase like 2 [Mus musculus]
CB489314	-1.01	-1.11	UNKNOWN
**CA769854**	**0.64**	**2.17**	**[GO] [P11404] Fatty acid-binding protein, heart (H-FABP) (Heart-type fatty acid- binding protein) (Mammary-derived growth inhibitor) (MDGI)**,
CB509453	-0.32	-0.48	[GO] [O16797] 60S ribosomal protein L3,
CB500821	-0.26	-0.36	[GO] [P62918] 60S ribosomal protein L8,
CB492885	-0.15	-0.31	UNKNOWN
CA061403	0.42	0.72	UNKNOWN
CB498219	0.24	0.51	[NR] [XP_613218] PREDICTED: similar to 24-dehydrocholesterol reductase precursor, partial [Bos taurus]
CA054168	-0.39	-0.58	UNKNOWN
CB515449	0.34	-0.60	[GO] [P50247] Adenosylhomocysteinase (EC 3,3,1,1) (S-adenosyl-L-homocysteine hydrolase) (AdoHcyase) (Liver copper binding protein) (CUBP),
CB515945	-0.36	-0.49	[NR] [NP_683732] RNA binding motif protein 5 [Mus musculus]
CA057448	-0.30	-0.33	UNKNOWN
CA036745	-0.62	-0.74	[NT] [XM_532501] PREDICTED: Canis familiaris similar to Chimerin (chimaerin) 2 (LOC475267), mRNA
CB509472	-0.29	-0.42	UNKNOWN
CB494192	0.23	0.33	[GO] [P09411] Phosphoglycerate kinase 1 (EC 2,7,2,3),
CB496589	-0.49	-0.82	UNKNOWN
CB513882	-0.48	-0.60	[NR] [XP_413822] PREDICTED: similar to normal mucosa of esophagus specific 1 [Gallus gallus]
CK990857	-0.64	-1.11	UNKNOWN

cDNAs or sets of cDNA putatively sensitive to estrogen exposure as judged by the presence on the top 250-lists ranked by moderated t-statistics on both 0.87 and 10 ng EE2/L exposure experiments in male juvenile rainbow trout. cDNAs corresponding to genes that were selected for qPCR analysis are marked with bold text. Note that several cDNAs may likely correspond to the same gene.

### Robust gene-expression changes

**Figure 2:**
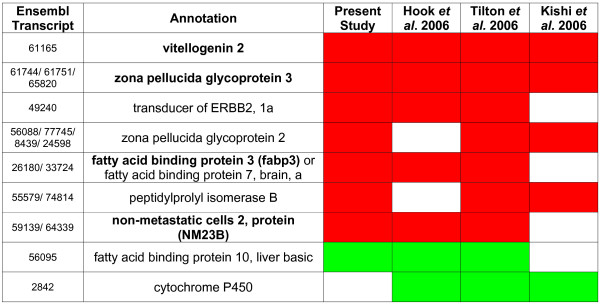
**Robust estrogen-responsive genes**. Genes affected by estrogen in at least three out of four studies used in the meta-analysis. Red refers to an up-regulation, whereas green refers to a down-regulation. Only the zebrafish transcripts with the best TBLASTX hit to each of the probes from the different studies are presented in the figure. Genes that were selected for qPCR analysis are marked with bold text.

A meta-analysis was performed with the aim to identify robust estrogen-responsive genes. The microarray data from fish (both sexes) exposed to 10 ng/L from the present study and available microarray data from three other exposure studies with fish and estradiol (E_2_) or EE_2 _were used in the meta-analysis [16,20,22]. Information about the different studies is shown in Table 2. Transcripts (360) presumably corresponding to 55 genes or groups of paralog genes were identified as differentially expressed in at least two of the four different studies (see Additional file 1). VTG and ZP3 were differentially expressed in all four studies and nine genes had an altered expression in at least three studies (Figure 2). It should be noted that ZP1 and the estrogen receptor-α, which are well-know estrogen-responsive genes in fish, have poor sequence representation on the cGRASP microarrays and are therefore not present in Figure 2.

**Table 2:** A summary of the four different studies used in the meta-analysis

	**Present study**	**Hook *et al*. 2006**	**Tilton *et al*. 2006**	**Kishi *et al*. 2006**
**Species**	*O.mykiss*	*O.mykiss*	*O.mykiss*	*O.latipes*
**Sex**	juvenile	male	juvenile	male
**Estrogenic substance**	EE_2_	EE_2_	E_2_	E_2_
**Exposure**	Water, 10 ng/L	Water, 50 ng/L	Dietary, 5 ppm	Water, 100 ng/L
**Water temperature (C°)**	10	12	12	24
**Duration (days)**	14	7	12	21
**Platform**	Two-channel spotted cDNA (GRASP 16 k v.1)	Two-channel spotted cDNA (GRASP 16 k v.1)	Two-channel spotted cDNA (GRASP 3.7 k v.1)	One-channel oligonucleotide (60 mer) (Kishi *et al*. 2006)
**Experimental Setup**	Direct comparison, 8 biological replicates	Direct comparison, 3 biological × 3 technical replicates	Reference design, 2 biological × 2 technical replicates	6 control and 3 exposed biological replicates
**Number of cDNAs/probes**	16006	16006	3700	22587
**Pre-processing**	Loess, no background correction	Loess	Loess	Robust Multichip
**Statistical method used for ranking**	Moderated t-statistic	Student t-statistic	fold change	Student t-statistic
**Selected cDNA/probes**	250 (167 induced, 83 suppressed)	189 (48 induced, 141 suppressed)	366 (127 induced, 239 suppressed)	381 (242 induced, 139 suppressed)
**Source for sequences**	cDNA, cGRASP	cDNA, cGRASP	cDNA, cGRASP	Transcripts, TIGR (OLGI release 4.0)
**Number of matches**^(*a*^**in *D.rerio ***	91	89	190	184

a) A match is defined as a tblastx hit with a E-value less than 10^-25^.

### Confirmation of microarray data with quantitative RT-PCR

Genes that were likely to be both sensitive (Table 1) and robust (Figure 2) were chosen for subsequent qPCR analysis. Three genes fulfilled these criteria: ZP3, a nucleoside diphosphate kinase (nm23) and fatty acid binding protein 3 (fabp3 or H-FABP). In addition, VTG was subjected to the qPCR analysis as well as the reference gene ubiquitin. In accordance with the microarray results the expression of VTG, ZP3 and nm23 were significantly induced in fish exposed to the high concentration. Also, as suggested by the microarrays, ZP3 and nm23 were significantly induced by the low concentration as well, whereas VTG expression was not induced (Figure 1). In stark contrast to the microarray results fabp3 had no tendency to any regulation caused by the treatment but showed a large variation within each treatment group (data not shown). The fabp3 and nm23 qPCR products were sequenced in order to confirm the amplification of the right products and they were identical to fabp3 and nm23 [EMBL:U95296, AF350241] in rainbow trout (data not shown).

## Discussion

Our meta-analysis correctly identified some of the most well known estrogen-responsive genes (VTG, ZP3, ZP2). This suggests that the approach has a good potential to identify other robust, less well known estrogen-regulated genes. We also showed that ZP3 and a hepatic nucleoside diphosphate kinase nm23 are more sensitive to estrogenic exposure than the widely used biomarker VTG. As far as we know, no other microarray study has identified the effects of as low concentrations of estrogen as used here. The recognition of nm23 induction as a highly sensitive response is therefore a novel finding. Thus we propose that analyses of nm23 together with ZP genes may increase the possibilities to detect an exposure to low levels of estrogenic compounds in fish. However, more studies are required in order to fully assess the potential of nm23 as a biomarker.

Sensitive biomarkers can be used as early warning signals to indicate exposure and thus potential risk of adverse effects. It has been suggested that the induction of ZP mRNAs are more sensitive than induction of VTG [9,23]. However expression of ZP genes can, as most genes, be affected by other environmental factors, for example cortisol exposure [10-12]. The regulation of a single gene is rarely sufficient to conclusively demonstrate a specific exposure, but a combination of responses would together potentially increase the degree of evidence.

We identified nine genes (or groups of paralog genes) that were affected by estrogen in at least three out of the four studies included in the meta-analysis. The known estrogen-responsive genes VTG and ZP3 were up-regulated in all four studies. The robust gene expression changes of ZP3, nm23 and fabp3 were also tentatively identified to be sensitive. However, the induction of fabp3 was not confirmed by qPCR. The incorrect identification from microarray data might be explained by cross hybridization to related mRNAs, a known problem for cDNA microarrays. Nm23, on the other hand, was confirmed with qPCR to be significantly induced both by a low and a high water concentration of EE_2_. In addition, microarray results from the other studies of rainbow trout exposed to 50 ng EE_2_/L and dietary exposed to 5 μg/g of E_2 _further supports an estrogen-induction of nm23 in rainbow trout during different exposure conditions [20,22]. The study of estrogen-exposed medaka did not report nm23 as an estrogen-responsive gene but it is unclear if nm23 was represented on the medaka microarray [16]. Whether nm23 is regulated by estrogen in other fish species is still an open question, although mammalian studies suggest a conserved induction mechanism [24].

Nm23 belongs to a larger class of nucleoside disphophate kinases that exist in multiple isoforms and are highly conserved throughout evolution. The investigated nm23 have been sequenced in rainbow trout [EMBL AF350241], Atlantic salmon [EMBL AF045187] and zebrafish [EMBL AF201764]. The salmon and zebrafish nm23 shows high similarity to the human nm23-H1 and H2 genes. A phylogenetic analysis suggests that nm23-H1 and H2 have arisen by gene duplication after the speciation event that gave rise to modern teleost fish and tetrapods [25]. Therefore it is assumed that the salmonid genome would only have one gene homologue to the nm23 -H1 and -H2 genes. In mammals the nm23-H2 gene encodes the c-MYC transcription factor and the nm23-H1 gene has been shown to be metastasis associated [24]. Moreover, the nm23-H1 gene and protein is up-regulated by E_2_-treatment in human breast carcinoma cell lines. This induction seems to be mediated, at least in part, at the transcriptional level via the estrogen receptor α binding to an estrogen responsive element in the promoter region of the human nm23-H1 [24]. The physiological function of nm23 in fish remains to be determined as well as the possibilities of a regulation by other factors than estrogen (specificity) and the robustness of the response during more complex exposure scenarios.

To be useful as a biomarker, a response should ideally be as robust as possible. In the meta-analysis we tested gene responses for robustness between species, exposure conditions and analytical platforms. Combining microarray data from different species and platforms is a challenging task, particularly when sequence information and good annotations are limited. We have addressed the cross platform/cross species comparison by using the zebrafish transcriptome as a reference. In contrast to most other fish species zebrafish has the advantage of being both well sequenced and well annotated. However, using zebrafish as a reference also has limitations, e.g. a lack of identified homologes for some genes. The results in the meta-analysis were also influenced by the shortage of available microarray raw data and therefore we had to accept the different statistical approaches used for selecting estrogen-responsive genes in the different studies.

It is certainly possible that more than nine hepatic genes are robustly regulated by estrogen in the analyzed species/conditions. We have only included comprehensive fish arrays in the meta-analysis. Nevertheless, many genes are still likely to be represented on only one or a few of the array platforms which limits the possibility of identifying robust responses. The choice of microarray platform also affects the possibilities to accurately identify differentially expressed genes. Amplicon arrays (cDNA arrays) show less concordance with other platforms, for example qPCR and commercially produced high density arrays with oligonucleotide probes or cDNA arrays with synthetic oligonucleotides [26]. Although, it has been shown that when two independent platforms give consistent results, the outcome of qPCR analysis will most often also be in agreement [27,28]. This adds confidence that several of the differentially expressed genes identified by the meta-analysis indeed are relatively robust responses.

By making the data on putative sensitive and/or robust gene responses public, it can be used as a base for further investigations on the effects of environmental estrogens in fish in order to develop biomarkers or to increase the understanding of the physiological impact of environmental estrogens.

## Conclusion

We have used microarrays to identify a range of potentially sensitive and/or robust gene expression changes in fish exposed to estrogen. We have identified the induction of ZP3 and a hepatic nm23 mRNA as being both sensitive and presumably robust responses. After further evaluation, nm23 induction would therefore be a good candidate biomarker together with ZP genes to reveal exposure to low levels of estrogens not sufficient to induce VTG but still with potential to affect gonadal differentiation of fish.

## Methods

### Experimental animals, exposure and preparation of hepatic total RNA

Fish from a previously published study were analysed [29]. The experimental setup was in short: 15, 14 and 14 juvenile rainbow trout (weighing around 100 g) were divided into three aquaria and exposed for two weeks to measured concentrations of 0, 0.87 and 10 ng/L respectively of EE_2 _in a flow-through system. Water samples were taken from the low and high EE_2 _concentration aquaria, before the transfer of the fish, on day 8 and on day 13. One sample was collected from the control aquaria on day 8. Solid phase columns were used to extract and purify EE_2 _from the water followed by derivatization (pentafluorobenzoylester) and further purification. EE_2_-concentrations were determined using GC/MS. The limit of detection (signal-to-noise set to 5) was 0.01 ng/L. Samuelsson *et al *showed that the fish exposed to 10 ng/L EE_2 _had significantly increased plasma levels of VTG, increased hepatosomatic index and the plasma metabolite profile were affected by the treatment. However, in the fish exposed to 0.87 ng/L neither induction of plasma VTG protein nor an altered metabolite pattern could be demonstrated using a specific VTG-ELISA and NMR respectively [29]. Gene responses in liver are widely used as biomarkers for environmental pollutants, i.e. estrogens, and the hepatic responses to estrogens are not restricted to a short developmental period. A prerequisite for our meta-analysis was availability of additional array-data from the same tissue in estrogen-exposed fish. Only hepatic microarray data was available in the literature, which therefore also contributed to our choice of tissue. Livers were collected and snap frozen in liquid nitrogen. Total hepatic RNA was isolated from individual trout liver using TRI reagent (Sigma chemicals Co, St Louis, MO, USA). RNA quality and quantity were assessed by agarose gel electrophoresis and spectrophotometric measurements (Nanodrop 1000, NanoDrop Technologies, USA and Spectra MAXplus, Molecular Devices, CA, USA).

### Microarray chip, hybridisation and wash

Salmonid cDNA microarrays (GRASP16k v1) were purchased from cGRASP, Univerity of Victoria, BC, Canada [21]. Microarray fabrication and quality control have previously been described in von Schalburg *et al*. [30]. The array contains 13,421 Atlantic salmon (*Salmon salar*) cDNAs and 2,576 rainbow trout cDNAs that together with a few more expressed sequence tags (ESTs) from other salmonid fish results in 16 006 spotted cDNAs in total. It has previously been shown that the sequence similarity between the Atlantic salmon and rainbow trout is sufficiently high for cross species use of the array [31].

Several cDNAs on the array correspond to the same gene and to reduce redundancy, a sequence based clustering was made as follows. Each cDNA sequence was compared to all other sequences on the array using BLAST [32]. A stringent cut-off value of at least 98% sequence similarity over 250 base-pairs or more was used to define equality. Single-link clustering was then applied which resulted in 13853 sets of cDNAs.

Slide preparation have been described in detail in von Schalburg *et al*. [30]. Briefly, 8 μg of total RNA was reverse transcribed and labelled using SuperScript Indirect cDNA labelling System kit (Invitrogen, Carlsbad, CA, USA) and fluorescent dyes Cy5 and Cy3 (GE Healthcare, Buckinghamshire, UK). cDNA from one control fish and one exposed fish were labelled and hybridised to the same array. Every other pair was dye swapped to compensate for cyanine flour effects. Eight male control fish were paired with male fish exposed to 0.87 ng EE2/L matching individual weights and lengths as closely as possible. Four of the same male control fish were also paired to fish exposed to 10 ng EE2/L. In the same way, four female control fish were paired both to females exposed to the low dose and the high dose. Hybridisation and wash were performed as described before by von Schalburg *et al*. [30] with the exception of the prehybridization that was preformed for 1.5 h in 5xSSC, 0.1% SDS, 0.2% BSA at 49°C. In total 20 microarrays were analysed.

### Microarray analyses

Fluorescent images of hybridized arrays were acquired using an Agilent MicroArray Scanner (Agilent Technologies, Palo Alto, CA, USA). Intensity data were extracted from TIFF images using Imagene version 6.0 (BioDiscovery, CA, USA). The statistical analysis was performed using the R package [33] LIMMA [34], which is available at the Bioconductor repository [35,36]. For each cDNA on the chip, M-values (log_2 _fold change) and A-values (average log_2 _intensity) were calculated. Loess normalization was applied to each array to remove intensity dependent trends [37]. For each set of cDNAs (defined above), an M-value was calculated by taking the average of the M-values of all the cDNAs in the set. Next, each set was annotated based on the cDNA with highest A-value (i.e. the spot with best hybridization). Finally, the sets of cDNAs were ranked by moderated t-statistic [34] to reduce the proportion of false positives. Data from the complete microarray experiment is available according to the MIAME guidelines at Array Express [38].

### Meta-analysis

Microarray data from four different studies on estrogen-exposed fish were subjected to a meta-analysis with the aim to identify robust estrogen responsive genes [16,20,22]. Another study with estrogen-exposed adult female zebrafish was excluded since the control fish presumable had high levels of endogenuous estrogen (neither VTG nor ZP3 was regulated in this study) [39]. To our knowledge, no other relevant microarray studies covering more than 3000 cDNAs/transcripts were publicly available (i.e. open access to transcript sequences) in October 2006 when we performed the meta-analyses. The studies included are summarized in Table 2.

The meta-analysis was done as follows. For each study, a list of the reported estrogen-regulated genes and the corresponding transcripts/cDNA-sequences was created. Note that the studies used different statistical methods to find the regulated genes (Table 2).

From the present study, the topmost 250 sets of cDNA from fish (both female and male) exposed to 10 ng EE2/L were chosen. To compare the platforms, zebrafish was used as a reference species. It was chosen since it is almost fully sequenced and well annotated compared to the other species involved. All transcripts/cDNAs were compared to the zebrafish transcriptome available through Ensembl release 40 (26,679 in total) using tblastx [32]. A cut-off E-value of 10^-25 ^was used to define a match. This resulted in a list of 360 zebrafish transcripts that had a match to transcripts/cDNAs from at least two studies (the transcripts should be regulated in the same direction). The list of zebrafish transcripts contained both multiple transcripts from the same gene (different splice variants) and paralogs and therefore the list was clustered into groups of transcripts. A similarity indicator matrix was created by comparing all transcripts in the list to each other using tblastx. Pairs of transcripts with an E-value of 10^-50 ^or less were defined to be equal. Otherwise the distance was set to zero. Single link clustering was then applied to create the groups of transcripts. Finally, all transcripts were annotated using Ensembl. The complete list of transcripts is available in Additional file 1.

### Quantitative RT-PCR

To confirm regulation of four selected genes, the abundances of the mRNAs were analysed with qPCR. The qPCR was performed on isolated total RNA from the same fish used in the microarray analysis. Total RNA (0.5 μg) was reverse transcribed in duplicate with a mixture of random hexamers and oligo(dT) primers, using the iScript™ cDNA Synthesis Kit (Bio-Rad, Hercules, CA, USA). The cDNA synthesis was performed according to the manufacturer's instructions, except for a scale-down of the reaction volume to 10 μl. Pooled RNA samples were used as no reverse transcriptase controls to control for genomic contamination. It was discovered that three samples might have been contaminated with DNA. These samples were treated with DNase and new qPCR analyses were done. PCR primers for ZP3 [EMBL:AF231708], nm23 [EMBL:AF350241], fabp3 [EMBL:U95296], VTG [EMBL:X92804], β-actin [EMBL:AJ438158] and ubiquitin [EMBL:AB036060] were designed using Primer3 software [40]. Primer sequences were as follows: 5'-ccctgcgtatctttgtgga-3' and 5'-gtgggaacctgtcattttgg-3' for ZP3; 5'-ccttcttccctggtctcgt-3' and 5'-gatgatgttcctgcccactt-3' for nm23 ; 5'-ctttccctgtttcccctcct-3' and 5'-tgctgtgtgcttcttgctactc-3' for VTG; 5'-ggggcagtatggcttgtatg-3' and 5'-ctggcaccctaatcacctct-3' for beta actin; 5'-cgatagacggtggtaagatgg-3' and 5'-aggtgtggcaaagggtagtg-3' for fabp3 ; 5'- atgtcaaggccaagatccag -3' and 5'-ataatgcctccacgaagacg -3' for ubiquitin. For all genes except the reference gene, ubiquitin (for which the qPCR was performed according to a previously published protocol [41]), the qPCR reactions contained 1× Real Time PCR Buffer, 3 mM MgCl_2_, 400 μM dNTP, 300 nM of each primer, 1 U TaKaRa Ex Taq™ R-PCR Version 2.1 (TaKaRa Bio Inc., Shiga, Japan), 0.25× SYBR Green I (Molecular Probes Eugene, OR, USA) and cDNA corresponding to 20 ng total RNA, in a final reaction volume of 20 μl. Real-time qPCR was performed on a Stratagene Mx3005p with 30 sec initial denaturation at 95°C, followed by 45 cycles of 95°C for 20s, 60°C for 20s and 72°C for 20s. A melting curve analysis was performed after each run to verify specific amplification. In addition the qPCR products were subjected to an agarose gel electrophoresis to confirm the expected size of the product. Both beta actin and ubiquitin were chosen as potential reference genes. Beta actin had a high variance and also a tendency to be regulated in the high dose group and therefore only ubiquitin was used. All signals were normalized against ubiquitin and ratios were calculated for exposed fish compared to control fish paired in the same manner as in the microarray analysis. Paired single-sided student's t-test were performed to test for significantly regulated genes. Since all samples could not be run at the same occasion, two standard samples were run at all occasions in order to enable a compensation for a possible run to run variation. Applying a run to run factor made little difference and the differently expressed genes VTG, ZP3 and nm23 were significant up-regulated both with and without applying the factor. The presented qPCR results are calculated without this factor.

## Authors' contributions

LG participated in the planning of the study, the sampling of the fish, carried out the molecular biology, participated in the statistical analysis, in the meta-analysis and drafted the manuscript. EK performed the statistical analysis and the meta-analysis and participated in the drafting of the manuscript. LF participated in the planning of the study. ON supervised the statistical analysis. DGJL planned the study, participated in the sampling, supervised and drafted the manuscript. All authors read and approved the final manuscript.

## Supplementary Material

Additional File 1 Transcripts or groups transcripts identified as differentially expressed in at least two of the four different studies Click here for file
